# Pollinaria Reconfiguration Mechanism of Widespread Euro-Mediterranean Orchids: The Effects of Increasing Air Temperature

**DOI:** 10.3390/plants11101327

**Published:** 2022-05-17

**Authors:** Micaela Lanzino, Anna Maria Palermo, Giuseppe Pellegrino

**Affiliations:** Department of Biology, Ecology and Earth Sciences, University of Calabria, 87036 Rende, Italy; anna_maria.palermo@unical.it (A.M.P.); giuseppe.pellegrino@unical.it (G.P.)

**Keywords:** orchids, pollinarium, pollinators, pollinaria bending, pollinaria reconfiguration

## Abstract

Orchids are fascinating for many reasons: their reproductive strategies, their pollination systems and the various morphological adaptations they have evolved, including the presence of pollen grains agglomerated into two masses, called pollinia, which form a structure known as a pollinarium. After withdrawal from a flower, the pollinarium undergoes a bending movement such that the pollen masses become correctly orientated to strike the stigma. We evaluated the duration of pollinator visits to inflorescences and the effects of temperature on pollinaria reconfiguration in eight orchid species in order to analyze the effects of increasing air temperature on the changes in bending time, and thus on geitonogamy levels. The impact of temperature on insect behavior was not assessed because our priority was to understand the effects of temperature on the process of pollinaria reconfiguration. All the examined species showed natural reconfiguration times that were 1.7–3.0 times longer than the pollinator residency times. A higher temperature showed a reduction in bending time regardless of the species tested. However, the bending time was never shorter than the residence time of the insects on the flower. Our data showed that high temperatures had a limited effect on the pollinarium reconfiguration time, thus indicating that high temperatures had a limited effect on folding compared to the effect that it had on the viability of the pollen.

## 1. Introduction

**Figure 1:**
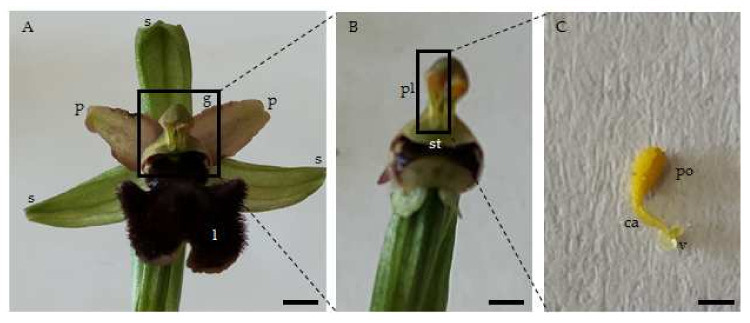
Flower (**A**), column (**B**) and pollinarium (**C**) of an orchid. P = petal, s = sepal, l = labellum, c = column, st = stigma, pl = pollinarium, po = pollinium, ca = caudicle, v = viscidium. Scale bar: 1 cm.

The Orchidaceae is a very large plant family of 32,000 species that is widely distributed across all continents [1] and occupies different habitats, from alpine to tropical and pluvial areas. A decisive factor in the survival of orchids is their interactions with other organisms. Due to their relatively small seeds, which lack the fundamental elements necessary for reproductive purposes (cotyledons and endosperm), these plants need mutualistic interactions with mycorrhizae for seed germination; while for successful pollination, orchids have developed various strategies for securing pollinator visits. The orchid flower has three sepals and three petals (including one called the labellum) (Figure 1A). Unlike most flowers, the male and female parts are combined into the column (Figure 1B). To compensate for the small number of visits by insects [2], orchid pollen is agglomerated into a unique mass, called the pollinium, which may contain more than a million pollen grains [3]. The pollinium, caudicle and viscidium form the pollinarium (Figure 1C). The pollinarium remains attached to the body of insects, especially hymenoptera, flies, wasps, solitary bees and bumblebees during a single visit, facilitating transport among flowers [4]. Many insects are attracted by the smell and color of the flowers and are rewarded by the presence of nectar. However, in several species of orchids, insects are attracted to deceptive visual cues, i.e., flowers without a nectar reward morphologically imitate those that provide one, influencing insect visits and therefore ensuring sexual reproduction via the deposition of pollinium from another flower [4]. Thus, orchids have different and specific pollination strategies; many genera are food-deceptive, i.e., *Orchis* L., *Anacamptis* Rich. and *Dactylorhiza* Neck. ex Nevski [4], all display flowers resembling those of rewarding orchids. *Ophrys* L. is sexually deceptive, showing flowers that mimic the female of its own pollinator in both shape and scent [5], and *Serapias* L. is shelter imitation deceptive, displaying flowers which offer refuge for insects to rest or sleep [6]. In addition to cross-pollination (mentioned above), which requires an insect as a vector, orchids show autogamy or geitonogamy. Self-pollination entails many disadvantages, such as genetic defects, a lack of variation, an inability of the plant to adapt to climate change, physical depression of the plant, a reduction in the pollen available to export to other plants and a significant increase in the rate of embryonic abortion [7]. A strategy for avoiding self-pollination is enacted by the reconfiguration mechanism of the pollinaria; this physical folding can reduce the likelihood of self-pollination. Already Darwin in 1878 had suggested that the physical reconfiguration of pollinaria serves as a mechanism for reducing the likelihood of self-pollination. Three pollinaria reconfiguration types have been identified in orchids, involving: pollinaria that shrink gradually to the correct size to be inserted into the stigmatic cavity [8], anther-caps that cover the pollinaria for a period following the pollinarium’s removal [9], and bending or twisting of an accessory structure (such as a stipe or caudicle) that connects the pollinium to a sticky pad (the viscidium). Bending is the mechanism by which the pollinaria, after their removal and before their insertion within the stigma, undergo a change in their orientation, flexing gradually [10]. Bending is caused by the rapid dehydration of the part by which the caudicle is connected to the viscidium [11]. The time it takes for the pollinarium to reorient varies widely in different species: it ranges from a few seconds (20–30 s) in *Orchis spitzelii* [12] or *Neotinea ustulata* [7] to several minutes in *Coeloglossum viride* [13] or *Ophrys insectifera* [14]. Through the physical folding of pollinarium, the correct position that allows the pollinium to touch the stigma is delayed until the pollinating insects leave for another plant. Obviously, in order for bending to reduce the likelihood of self-pollination, it is necessary that this process take place for a longer time than the duration of the pollinator’s visit. Peter and Johnson [7] showed that there is a strong positive relationship between the reconfiguration time and the duration of pollinator visits. Reconfiguration times were also consistently longer than pollinator visit times. In general, the bending process was not completed until after 1.5 times the mean period spent on an inflorescence by a pollinator [7]. Since air temperature variations could change the folding time of the pollinarium [11], we wondered: how can we evaluate the effects of temperature on bending? In the last few decades, much attention has been paid to the impact of climate change on orchid biology. It has been shown that an increase in temperature has a strong influence on orchid distribution, reducing the availability of suitable habitats [15,16], and on pollen viability duration, reducing the viability of pollen and consequently, the reproductive success of the plants [17]. The objectives of this study were to expand upon the current information concerning the pollinaria reconfiguration mechanism of widespread Euro-Mediterranean orchids; investigate the effects of increasing air temperature on pollinaria reconfiguration time; collect observations of orchid pollinators and their foraging behaviors on inflorescences with particular attention to the permanence times of the insect on the flower.

## 2. Results

**Figure 2:**
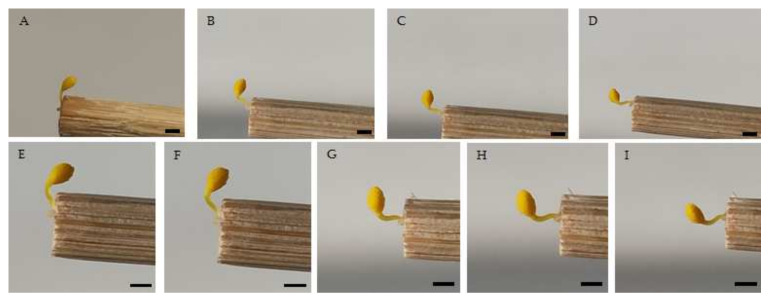
Photos (5×) of the bending movement of pollinaria. *Ophrys sphegodes* (**A**) just removed with toothpicks, (**B**) after 30 s, (**C**) after 90 s and (**D**) after 160 s; *Dactylorhiza sambucina* (**E**) just removed with toothpicks, (**F**) after 10 sec, (**G**) after 15 s, (**H**) after 25 s, and (**I**) after 35 s. After photo (**D**,**I**) no further bending movements were observed. Scale bar: 1 cm.

The pollinaria reconfiguration mechanism of all examined orchids was neither anther cap retention nor pollinium shrinking, but bending, similar to the more common reconfiguration type found in most other orchids [18]. The bending movement was localized at the point at which the caudicle joined the viscidium. The pollinarium pulled out of the thecae manually were projected almost perpendicularly on the tip of the toothpick at first, and then they bent forward through an arc of 30–120° (Figure 2).

**Figure 3:**
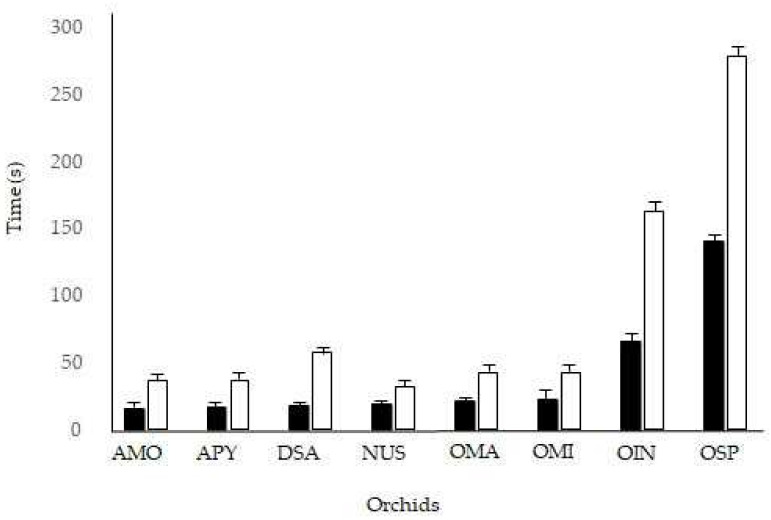
Pollinator residency time (black bars) and pollinaria reconfiguration time (white bars) of examined orchids in the field. AMO = *Anacamptis morio*, APY = *A. pyramidalis*, DSA = *Dactylorhiza sambucina*, NUS = *Neotinea ustulata*, OMA = *Orchis mascula*, OMI = *Orchis militaris,* OIN = *Ophrys insectifera*, OSP = *O. sphegodes*.

Under natural conditions, the pollinaria reconfiguration took from 30 s in *D. sambucina* to 310 s in *O. insectifera*; the pollinator residency time ranged from 12 s in three orchid species to 158 s in *O. insectifera* (Table 1). The mean reconfiguration time of a species’ pollinaria was positively related to the mean time that pollinators spent visiting a single inflorescence. Specimens belonging to genera *Ophrys* showed higher values for pollinaria reconfiguration times and pollinator visit times, at least four times longer than other species (Table 1). The field observations highlighted that all the examined species showed reconfiguration times that were 1.7–3.0 times longer than pollinator residency times (Figure 3).

**Figure 4:**
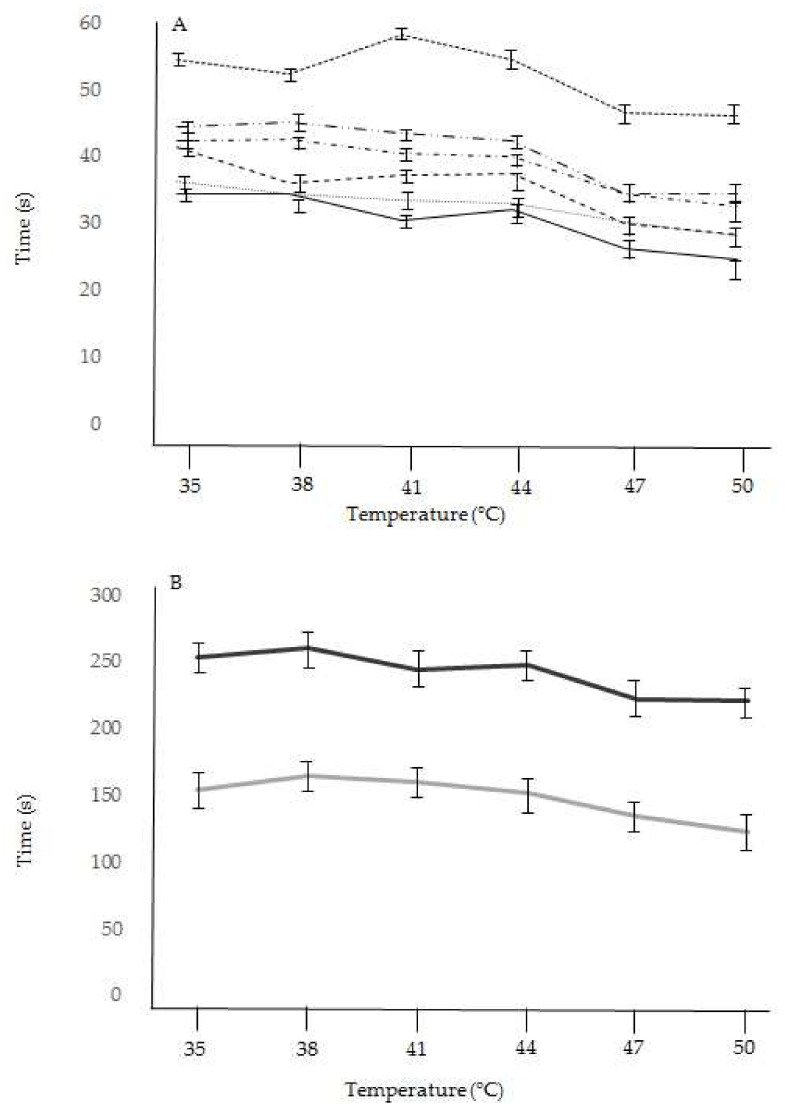
Reconfiguration time of pollinaria stored at high temperatures (35–50 °C) of food-deceptive orchids (**A**) and sexually deceptive orchids (**B**).

**Figure 5:**
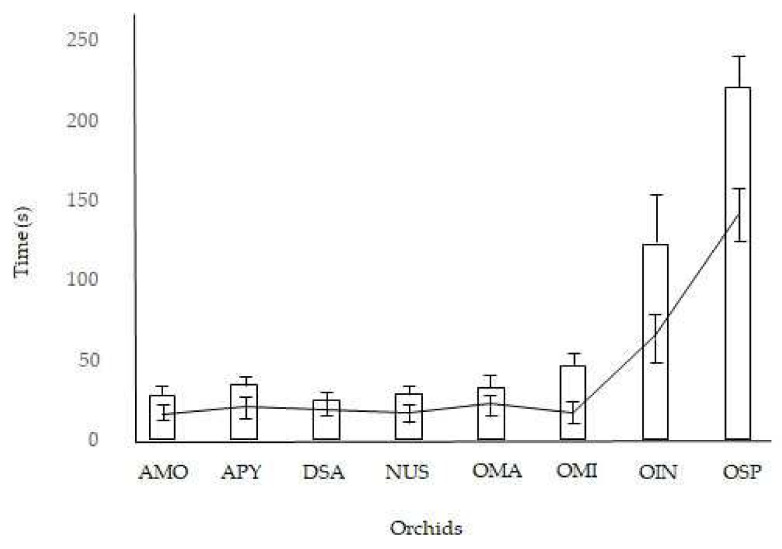
Pollinator residency time (black line) and reconfiguration time of pollinaria stored at 50 °C (white bars) of examined orchids. AMO = *Anacamptis morio*, APY = *A. pyramidalis*, DSA = *Dactylorhiza sambucina*, NUS = *Neotinea ustulata*, OMA = *Orchis mascula*, OMI = *Orchis militaris,* OIN = *Ophrys insectifera*, OSP = *O. sphegodes*.

The pollinaria of all examined flowers that were stored in an oven at 35–44 °C showed no significant reduction (F_30,264_ = 0.644, *p* = 0.32), or a reduction of less than 10%, in the bending time compared to pollinaria under natural conditions (Table 2, Figure 4). Pollinaria subjected to higher temperatures of 47–50 °C showed a significantly greater decrease in their reconfiguration times, ranging from 10% to 25% (Table 2, Figure 4) of the natural value (F_22,264_ = 0.042, *p* = < 0.001). There were no significant differences among species in the percentages of the reduction of the bending time for pollinaria stored at a different temperature (F_15,264_ = 0.712, *p* = 0.49). In every case, the bending time of each species was never shorter than the residence time of the insects on the flower. However, in some species, in the experiments at higher temperatures, the pollinaria reconfiguration time and the pollinator visit time were very close. In particular, the reconfiguration time of the pollinarium of *D. sambucina*, *Neotinea ustulata* and *Orchis mascula* stored at 50 °C was very close to the maximum value of the pollinator visit time (Figure 5). However, the bending times measured at high temperatures were still longer than the residence time measured under natural conditions.

## 3. Discussion

Field observations contained in this study highlight the fact that pollinators spend less time on a single orchid than the time taken for a pollinarium to undergo a curving movement, reducing the possibility of the occurrence of self-pollination among flowers on the same plant. Our results agree with Darwin’s intuitive interpretation of bending [18] when he suggested that the slowness of pollinarium folding is a mechanism to prevent geitonogamy events. Subsequent studies [16,18] have highlighted how Mediterranean orchids show a relationship between the times that pollinators spend on an inflorescence and the time of a complete bending movement. For instance, the pollinators of *Orchis mascula* spend about 10 s visiting orchid inflorescence, while pollinarium need at least 30 s to undergo bending after withdrawal [19]. In our case, pollinators spend no more than 28 s on *O. mascula* flowers, while pollinaria reconfiguration times range from 40 to 46 s. The reconfiguration mechanism of the species of *Eulophia* takes from 82 s to 155 s, while the duration of the observed visits to inflorescences of pollinators, notably the *Xylocopa* or *Megachile* species, lasted less than 1 min [18].

Temperature increases have adverse effects on various aspects of orchid reproduction. Many deceptive orchid species modify the start date of flowering [20] or show a shift in their phenology [21] due to an increase in air temperature. 

The reproductive success of orchids is closely linked to their interaction with pollinating insects, and the spectrum of pollinators may change. The drastic effects of the variations of plant/insect mutualism are more evident for orchids that show a species-specific relationship with a pollinator. Hutchings et al. (2018) [22] showed that populations in England of sexual deceptive *Ophrys sphegodes* achieved limited pollination success or complete reproductive failure because global warming modified the emerging time of the pollinators, namely, male *Andrena nigroaenea* bees. 

However, an important role for the reproductive success of plants is dependent on pollen and its viability. Orchid pollen is agglomerated into dispersal units [3], which remain attached to the body of the pollinator when the insect rests on the orchid labellum. For successful fertilization, the pollen must remain viable for many consecutive days, and therefore, it is important that it has a certain longevity and viability after long-term preservation [23]. The lifespan of pollinarium has been reported to range from a few minutes to several days in orchid species [24,25]. High temperatures have dramatic effects on the viability of pollen. Previous studies highlighted that pollinaria stored at 41–44 °C for 2–4 days showed a drastic reduction in pollen viability, and under more severe conditions, 47–50 °C, any possible pollen germination was prevented [16]. 

Our data show that high temperatures have limited effects on the pollinarium reconfiguration time. In all examined species, the reduction of the bending time is never shorter than the residence time of the insects on the flower of the same species.

Temperature affects not only several aspects of an orchids’ life (such as survival and distribution), but also the behavior of the insects. In this study, however, our priority was not to focus on the effects of temperature on insect behavior, but instead, the main goal of the project was to understand how temperature affects the bending mechanism of orchids. In any case, the data in the literature show that warmer temperatures did not directly modify insect behavior in terms of visiting time but decreased floral nectar production, and bumblebees visited flowers suffering from nectar reduction four times less frequently than they visited those plants with a natural concentration of nectar [26]. In our case, the examined orchids were no nectariferous and so they could not have any modifications of floral reward due to higher temperatures. Moreover, bees and butterflies prefer warmer temperatures than other hymenopterans [27]. These data suggest that bending remains a valid physical mechanism for promoting cross-pollination even at high temperatures, reducing the likelihood of self-pollination. 

It should be added, however, that for some orchid species that were examined in this study, the average bending value at 50 °C was very close to the maximum time that an insect spends on the flowers of the same plant. This implies that geitonogamy events can occur in some cases. The bending times measured at high temperatures were still longer than the residence time measured under natural conditions.

It has been shown that there is a relationship between the visiting time of insects and orchid pollination strategies. Primarily, insects visit more flowers and forage longer on the inflorescences of nectar-producing orchids [4]. Field experiments in which the flowers of nectarless orchids were supplied with complementary nectar showed that insects increased their residence time on the inflorescence compared to natural conditions [28], increasing geitonogamy [29]. Probably the shorter time taken by insects visiting nectarless orchids rather than rewarding orchids reveals a higher probability of cross-pollination in the deceptive species than in the nectariferous species [30]. This implies that pollinators visit fewer flowers more quickly in sequence in the deceptive species, which gives the pollen a high chance of being exported to another plant, reducing self-pollination events [4]. Our data support a relationship between pollinaria bending time and pollination strategies. The sexually deceptive orchids showed a higher value for their reconfiguration time than did the food-deceptive orchids, probably due to the difference in pollinator visiting times. The pseudocopulation strategy of *Ophrys* prompted insects to visit more flowers consecutively on the same plant, while pollinators spent less time on food-deceptive orchids [4,31]. The delayed pollinaria bending mechanism limited the events of geitonogamy, and the average rate of self-pollination tended to be higher in the rewarding orchids than in the deceptive orchids [32]. In any case, the data from the literature show that the bending mechanism limits but does not completely exclude the possibility of geitonogamy in deceptive orchids [33,34,35]. 

The effects of rising temperatures have a marginal effect on bending movements compared to the effects observed concerning the viability of the pollen [17]. Certainly, the lack of pollen germination has consequences that are more drastic than the effects of a reduction in bending time on reproductive success. While a rise in temperature can lead to the absence of viable pollen and therefore drastically reduce reproductive success, pollinaria reconfiguration modifications can cause an increase in self-crossing. In the first case, we have a reduction in the reproductive success of the plant, in the second, only a reduction in the percentage of cross-pollination. Therefore, an increase in temperature significantly reduces pollen viability [17], but it has no dangerous effects on bending. 

## 4. Materials and Methods

### 4.1. Plant Material

Eight species of the genera *Anacamptis*, *Dactylorhiza*, *Neotinea*, *Ophrys* and *Orchis*, all belonging to the subtribe Orchidinae, were selected (Table 3) from Calabria, southern Italy. Calabria has a highly variable climate, strongly influenced by the presence of the sea and of the mountains. The climate type of the coastal areas is a Mediterranean climate, characterized by hot, dry summers and cool, wet winters; the interior mountain areas have a typical mountain climate, with snow during winter. All the examined orchids were food-deceptive except *Ophrys*, which uses a sexual deception strategy. These species were chosen since they did not show a different morphology of pollinaria in terms of the length of the caudicle or the thickness of pollinium (personal morphometric analysis).

### 4.2. Pollinaria Reconfiguration Time

To test spontaneous pollinaria reconfiguration during the spring of 2019, the pollinaria of ten flowers from two populations of each of the examined orchid species were carefully removed with toothpicks, and then photographs were taken every 5–10 s until further folding movements were observed. The air temperature was noted, and observations were made only if the recorded temperature was between 28 and 35 °C. This temperature range was chosen considering the monthly and annual temperature datasets of the Centro Funzionale Multirischi of Calabria Region (http://www.cfd.calabria.it/ (accessed on 1 April 2019) for the period 1916–2010 [36].

### 4.3. Air Temperature Effects

To evaluate the effects from air temperature on pollinaria bending, during spring of 2019, five plants with at least 75% unopened flowers from two populations of each of the selected species were transferred to a green house. Pollinaria were removed using toothpicks from three flowers for each specimen. The toothpicks carrying the pollinaria were fixed on a piece of Styrofoam at different temperatures—35 °C, 38 °C, 41 °C, 44 °C, 47 °C and 50 °C—in an oven with a glass door. We chose 35 °C as the starting temperature, as it is the temperature at which we carried out the field observations, and 50 °C because it is close to the highest temperature recorded in the areas studied (45 °C, July 1974, 1993, 2007, http://www.cfd.calabria.it/ (accessed on 1 April 2019). Six pollinaria from each temperature class for each species were tested. Incidents of the bending of orchid pollinaria were recorded with a digital camera. We considered it to be the end of the folding time when there was no longer any movement of the pollinaria. 

### 4.4. Pollinator Observation

To ascertain the duration of pollinator visits to inflorescences, during the peak of the flowering period, five flowering individuals in each population whose flowers contained pollinaria were videotaped using digital video cameras (Kodak camera Zi8) during four sunny, warm (air temperature between 30–35 °C), and windless days (wind speed below 5 Km/h) between 10:00 a.m. and 4:00 p.m. The camera was placed on a tripod about 1.5 m away from the plants; 24 h of observation in each population were conducted for a total of 384 h of field observation. The recordings were analyzed, and we recorded the time of the pollinators on a single flower and on an inflorescence.

### 4.5. Data Analysis

The effects of the pollinaria reconfiguration time were evaluated using an analysis of variance (ANOVA) with taxa as fixed factors, using the SPSS software package (SPSS v. 13·0 for Windows, Chicago, IL, USA). In particular, we compared pollinaria reconfiguration time and pollinator residency time, and pollinaria reconfiguration time and air temperature among the eight species. Moreover, bivariate analyses were performed using the SAS package (SAS Institute, Inc., Cary, NC, USA, 1988) to evaluate the effects of air temperature on the pollinaria reconfiguration time. 

## Figures and Tables

**Table 1:** Reconfiguration times for orchid pollinaria and the pollinator residency time on orchid inflorescence under field conditions (air temperature between 30–35 °C). n = sample size.

Taxon	Reconfiguration Time (s) n = 160			Pollinator Visit Time (s) n = 80		
	min	max	mean ± SE	min	max	Mean ± SE
*Anacamptis morio*	35	38	36.5 ± 1.2	12	20	15.6 ± 2.6
*Anacamptis pyramidalis*	40	45	43.2 ± 1.7	16	28	21.2 ± 3.7
*Dactylorhiza sambucina*	30	35	32.5 ± 1.9	15	22	18.9 ± 2.6
*Neotinea ustulata*	35	40	37.3 ± 2.1	12	22	17.0 ± 3.0
*Ophrys sphegodes*	150	172	163.3 ± 7.4	50	82	65.6 ± 9.8
*Ophrys insectifera*	250	310	279.1 ± 19.6	120	158	141.1 ± 10.6
*Orchis mascula*	40	46	43.0 ± 2.5	18	28	22.6 ± 3.5
*Orchis militaris*	55	62	57.8 ± 2.4	12	24	17.5 ± 3.7

**Table 2:** Mean ± SE of reconfiguration times (sec) for orchid pollinaria stored at different temperatures. n = sample size.

Taxon (n = 240)	Temperature					
	35 °C	38 °C	41 °C	44 °C	47 °C	50 °C
*Anacamptis morio*	36.2 ± 1.2	34.4 ± 1.1	33.5 ± 1.3	33.0 ± 1.2	30.1 ± 1.2	28.2 ± 1.4
*Anacamptis pyramidalis*	44.5 ± 1.6	45.2 ± 1.6	43.5 ± 1.7	42.3 ± 1.5	34.4 ± 1.6	34.3 ± 1.5
*Dactylorhiza sambucina*	34.5 ± 1.7	34.3 ± 1.5	30.5 ± 1.4	32.0 ± 1.6	26.3 ± 1.7	24.5 ± 1.3
*Neotinea ustulata*	41.5 ± 2.2	36.0 ± 1.9	37.3 ± 1.8	37.5 ± 1.8	30.0 ± 1.6	28.3 ± 1.4
*Ophrys sphegodes*	154.5 ± 5.2	164.3 ± 6.2	160.5 ± 5.5	152.0 ± 4.2	134.3 ± 4.8	122.5 ± 4.2
*Ophrys insectifera*	254.0 ± 11.2	260.5 ± 10.6	244.2 ± 10.2	248.3 ± 11.4	222.5 ± 10.9	220.5 ± 11.2
*Orchis mascula*	42.3 ± 2.2	42.5 ± 2.3	40.5 ± 2.1	40.0 ± 1.9	34.5 ± 2.2	32.5 ± 1.9
*Orchis militaris*	54.5 ± 2.3	52.3 ± 2.4	58.2 ± 2.1	54.5 ± 2.2	46.5 ± 2.1	46.2 ± 2.0

**Table 3:** Sampling locations, pollination syndromes and predominant pollinators of the studied orchid species.

Species	Sampling Location *	Pollination Syndrome	Predominant Pollinators
*Anacamptis morio*	Mangone	Food deception	Bumblebees
	Cupone		
*Anacamptis pyramidalis*	Acquaformosa	Food deception	Butterflies
	Cassano		
*Dactylorhiza sambucina*	Carlo Magno	Food deception	Bumblebees
	Botte Donato		
*Neotinea ustulata*	Petrosa	Food deception	Tachinid flies
	Firmo		
*Ophrys insectifera*	Cassano	Sexual deception	Wasps
	Petrosa		
*Ophrys sphegodes*	Piano Monello	Sexual deception	Sand bees
	Piano Lago		
*Orchis mascula*	Cupone	Food deception	Cuckoo bumblebees
	Cecita		and solitary bees
*Orchis militaris*	Rogliano	Food deception	Cuckoo bumblebees
	Frascineto		and solitary bees

* All locations are in Calabria, southern Italy.

## Data Availability

Not applicable.
